# The Impacts of *TRR14*-Overexpression on *Arabidopsis thaliana * Growth and Photosynthetic Parameters

**DOI:** 10.15171/ijb.1342

**Published:** 2017-03

**Authors:** Habib Rezapoor, Mahnaz Aghdasi, Hamid Reza Sadeghipoor

**Affiliations:** Department of Biology, Faculty of Science, Golestan University, Gorgan, P.O.Box: 159, Iran

**Keywords:** Growth, Overexpression, Photorespiration, Protein, TRR14

## Abstract

**Background:**

TRR14 protein is a small protein, a member of a multigene family in Arabidopsis which was found as the fi rst protein during screening seedlings for their resistant to the trehalose sugar.

**Objectives:**

A number of TRR14-overexpressing plants were subjected to the characterization in the present research, among which, the associated morphological features and changes accompany growth pattern and photosynthesis related parameters.

**Materials and Methods:**

*TRR14* gene was isolated from *Arabidopsis Thaliana* and cloned into the pBin-35S vector. Recombinant vector was transferred to the *Arabidopsis* (Col-0) via *Agrobacterium tumefaciens* using the Floral Dipping method. Seeds from the *TRR14* overexpressed (TRR14) and the Col-0 wild-type (WT) plants were shown on soil under long day conditions. Several measurements were then performed including determination of the fresh and dry weights, leaf area, chlorophyll a and b (Chl a and Chl b) content, Chl a/b ratio, total chlorophyll and carotenoids content, soluble and insoluble sugars content, total and soluble protein content, the Hill reaction rate, chlorophyll fl uorescence, as well as photorespiration rate. Meanwhile, the chloroplastic proteins were investigated by SDS-PAGE analysis.

**Results:**

TRR14 plants showed a signifi cant increase in fresh and dry weights, leaf area, and total and soluble protein content along with a signifi cant decrease in the insoluble sugar contents was observed in comparison to the WT plants. Chl a, Chl b, total chlorophyll content, Chl a/b ratio, carotenoids content, Hill reaction rate, and chlorophyll fl uorescence didn’t show a signifi cant diff erence between TRR14 and WT plants. The SDS-PAGE gel electrophoresis of the chloroplastic proteins showed a thick band with a molecular mass of 25 kDa in *TRR14*-overexpressed plants, compared to the WT plants. Remarkably, photorespiration rate was decreased in TRR14 plants compared to WT plants.

**Conclusion:**

The increased biomass of TRR14 transformed plants might be due to its ability in reducing photorespiration through concentrating CO_2_ in the leaf’s intercellular spaces.

## 1. Background

Encoded as *At4g10300*, TRR14 (Trehalose Resistance) protein is 139 aa in length and contains Cupin domain (1,2). In plants, proteins containing conserved Cupin domain play diverse functions in the metabolism and signaling. Enzymes such as: phosphomanose-isomerase, polyketide synthase, dioxygenase, oxalate oxidase (germins), and auxin binding protein belong to this group (2,3). So far 18 different functional Cupin subclasses were identified in plants (2). TRR14 has six paralogues in Arabidopsis and even more orthologous in *Oryza sativa*, and *Medicago sativa*. Beyond plants, the closest TRR14’s homologs are found in the bacteria, particularly in many cyanobacteria including *Synechococcus sp.* and *Prochlorococcus marinus*. This protein has been localized in the chloroplast and its expression is ubiquitous (4).

Trehalose metabolism has recently been recognized to play an important role in carbon signaling in the plants (5,6,7). Trehalose is the alpha, alpha-1-1 linked glucose disaccharide, which is found ubiquitously and is being thought to be evolutionary ancient (8). The synthesis of the trehalose-6-phosphate (T6P), the immediate precursor of the trehalose, is indispensable for development and carbon utilization in Arabidopsis seedlings (9). Meanwhile, T6P is a signal for sucrose status and regulate the content of sucrose in the plant (10,11). Plants overexpressing trehalose-6-phosphate synthetase (*TPS*) display an increased T6P and typically have dark green cotyledons, leaves, as well as an increased photosynthetic capacity per leaf area unit (12).

Photorespiration is an indispensable cleavage of ribulose 1, 5- bisphosphate (RuBP) by the molecular oxygen which leads to an equimolar production of 3-phosphoglycerate (3-PGA) and 2-phosphoglycolate (2-PG). This process occurs because Rubisco (i.e. the enzyme which catalyzes the carboxylation of RuBP) is also able to oxygenate this compound. While 3-PGA can easily be utilized by the Calvin cycle for the production of sugars, the two-carbon compound 2-PG is toxic and its removal from photosynthesizing cells requires a long metabolic pathway known as photorespiratory carbon cycle which is accompanied by the release of CO_2_ (13).

The Photorespiration-mediated CO_2_ release imposes yield loss on most of the highly bred crops plants, particularly when experiencing heat or drought stresses which favor CO_2_ shortage and O_2_ accumulations in photosynthesizing mesophyll cells due to stomata closure (14,15).

## 2. Objectives

TRR14 is a novel protein and its function is not precisely understood. Our previous results showed that TRR14 might be involved in tolerance of the plants toward salinity and drought stresses (4,17), as plants with the capacity to restrict their photorespiration are more efficient in tolerating these two type of stresses (17,18,19,20). In the present research, the impact of *TRR14* overexpression was evaluated on *Arabidopsis thaliana* with respect to several morphological and physiological parameters related to the photosynthesis and photorespiration. The results obtained from such studies may lead to a better understanding of the possible links between trehalose metabolism, photosynthesis, and photorespiration.

## 3. Materials and Methods

### 3.1. Wild-type Plants and Transgenic Lines’ Growth

Seeds from *Arabidopsis thaliana* wild-type (WT) plants ecotype Columbia-0 (COL-0) and transgenic lines (TRR14) were planted in compostsoil (Takidar-Iran). Plants were grown in the controlled growth chamber with a light normal irradiance of 150 μmol photon m-^2^s^-1^ in a light-dark cycle of 16/8 hours at 25°C.

### 3.2. RNA Extraction and cDNA Synthesis

Plant material was immediately frozen in the liquid nitrogen and was ground to the powder in a dismembrator (Braun, Melsungen, Germany). The total RNA was obtained using the RNeasy plant mini kit, according to the instruction of the manufacturer (QIAGEN USA, Valencia, CA). The quantity and quality of the extracted RNA were determined using spectrophotometeric method and agarose gel electrophoresis, respectively. Genomic DNA contamination was removed with DNAse I (DNA-free, Ambion, Austin, USA) treatment. The absence of DNA was analyzed by performing a PCR reaction (40 cycles) on the DNase *I*-treated RNA, cDNA synthesis, and PCR amplication using *Taq* DNA polymerase and specific primers as follows. cDNA synthesis was performed using M-MLV reverse transcriptase (Promega, Madison, WI) on 1ng of the total extracted RNA with 0.5 μg of oligodT16v (custom oligo from Invitrogen, Carlsbad, CA) and 0.5 μg of random hexamer (Invitrogen, USA). PCR was performed with forward and reverse primers (*TRR14F*: 5^’^- ACCCAACTCGGTGTTCGTAG-3^’^ and *TRR14R*: 5^’^-TGATAGCAGCCATTCACTAG-3^’^).

### 3.3. Plasmid Construction

The obtained PCR fragments were cloned into the pGEM-T Easy vector (Promega, Madison, USA) and sequenced. The fragment was then isolated and purified from the pGEM-T easy vector clones and cloned into the pBin19 expression vector using standard restriction/ligation techniques (*Hin*dIII/*Eco*RI). The CaMV 35S promoter was isolated by digestion with *Eco*RV from the pUC-18 vector and subsequently ligated into pBin19 (*Hin*dIII/*Eco*RI).

### 3.4. Plant Transformation

The construct was transformed into *Agrobacterium Tumefaciens* Gv3101 by using a Gene Pulster electroporator (BioRad, Hercules, Ca, USA), according to the manual. Then, *Arabidopsis thaliana* ecotype Col-0 plants were transformed by the floral dip method (21). Transgenic T1 seedlings were selected on half MS medium supplemented with the 50 mg.L^-1^ Kanamycin. A transformation with the full-length cDNA of TRR14 has produced 20 independent lines.

### 3.5. Quantitative Real Time PCR Analysis

Total RNA from overexpressed and the WT plants were isolated using RNeasy plant mini kit (QIAGEN USA, Valencia, CA). After cDNA synthesis, Quantitative-PCR (Q-PCR) was performed using ABI-prism 7700 Sequence Detection System (PE-Applied Biosystems, Foster City, CA) according to the standard SYBR green PCR Master Mix (Applied Biosystems, UK) and specific primers (*TRR14F*: 5^’^-AACTTGATCGGGATATGGAGTG-3^’^*TRR14R*: 5^’^-AACTTGATCGGGATATGGAGTG-3^’^). The Actin gene(*At3g18780*)was used to serve as house the keeping gene and the relevant primers for measuring its expression level were as following sequences: *AtACTIN2F:* 5^’^- GACCCAAAGACGGAGACTCTT-3^’^ and *AtACTIN2R:* 5^’^- GCCAAGTGATTGTGGAGACTC-3^’^. Each experiment was repeated 3 times. Gene expression was normalized to the expression of *AtACTIN2*as the calibrator reference gene, using the comparative Ct method (22). Melting curve analysis was done for checking the specificity of the PCR reaction and lack of primer dimers, as well. For further confirmation, PCR products were loaded onto a %2 agarose gel, ran, and visualized.

### 3.6. Pigment Content and Fluorescence Measurements

Chlorophylls a, b, total chlorophyll, and carotenoids’ contents were determined essentially as described by Jeffery and Humphrey (23). Leaves from the 3 weeks old plants were frozen in liquid nitrogen and ground in 80% (v/v) acetone. The absorbance was then measured at 670, 645, and 663 nm (Shimadzu UV-160). The chlorophylls a, b, total chlorophyll, and carotenoids’ content were then calculated according to the following formula:

C_a_= (12.76_663_-2.69A_645_)V/FW

C_b_= (22.9_645_-4.68A_663_)V/FW

C_total_= (20.26_645_-8.02A_663_)V/FW

C_carotenoid_= (1000A_470_-1.82C_a_- 85/02C_b_)/198

The fluorescence of Chlorophyll *a* was determined with OPTI-Sciences OS-30 fluorometer (Walz, Effeltrich, Germany). After 15 min adaptation of the Arabidopsis plants to the dark, F_0_ (the initial fluorescence content of PSII reaction center) was determined in the presence of 10 μmol photons m.s^-1^ measuring beam. The *F*m (maximum fluorescence content in the dark-adapted state) was determined using a 0.8 s saturating irradiance pulse. The fluorescence parameter *F*v*/F*m was calculated using the DualPAM software**.**

### 3.7. Chloroplast Preparations and Measurement of the Hill Reaction Rate

The rate of Hill reaction in the chloroplast preparations was determined according to the Trebst (24). Chloroplasts were extracted from the frozen ground leaf material in the ice-cold buffer consisting of 20 mM Tris-HCl (pH 7.5), 0.3 M sucrose, 10 mM EDTA and 5 mM MgCl_2_. The resulting crude extract was filtered and then centrifuged at 3500 ×*g* for 5 min. The obtained pellet was re-suspended in 10 mLof the ice-cold homogenate buffer thoroughly with a Pasteur pipet. The chloroplast suspension was stored on ice all the times.

The Hill reaction rate in the prepared chloroplast was determined spectrophotometrically by measuring absorbance at 600 nm due to the reduction of Dichlorophenolindophenol (DCPIP). The Hill reaction rate was expressed as the changes in absorbance per milligram chlorophyll per minute.

### 3.8. Carbohydrate and Protein Measurement

The soluble and insoluble sugars’ content was determined spectrophotometrically, as described by Kochert (25). The soluble and the total proteins of the leaf were determined according to the methods to the Bradford (26) and Markwell (27), respectively.

### 3.9. SDS-PAGE Analysis of the Chloroplast Proteins

The SDS-PAGE of the chloroplast protein samples was carried out according to Flingand Gregerson (28). The chloroplast preparation was suspended in the 100 μL of the homogenization buffer. After adding 5.0 mL n-Hexan:2-propanol (3/2; v/v), it was centrifuged at 4000 ×*g* for 15 min. Then the precipitated protein was washed with 5.0 mL acetone (80% v/v) and dried under a stream of the nitrogen gas. The dried protein precipitates were fractioned by SDS-PAGE using 15% w/v polyacrylamide gels that were subsequently stained with the Coomassie blue (R250, Sigma, Germany).

### 3.10. Photorespiration Measurement

Plants were grown in soil under a short day regime at 22^o^C. The setup was used to test the differences between the response of *TRR14* overexpressed and WT plants to the air with 2% oxygen content and room air with the normal 21% oxygen content. The ratios of the leaf internal versus air CO_2_ concentrations were measured in the WT and in the overexpressed *TRR14* plants. Photorespiration was calculated by using the following formula: (Vo+Rp)/(Vo+Vc), where Rp is the photorespiratory CO_2_ release, Vc the rate of carboxylation and Vo the rate of oxygenation (29).

### 3.11. Statistical Analyses

The data were analyzed by statistical SAS package (version 9). The reported values were means of the three replicates. The Duncan’s test was used to compare means for the significance.

## 4. Results

### 4.1. Overexpression of TRR14

**Figure 1:**
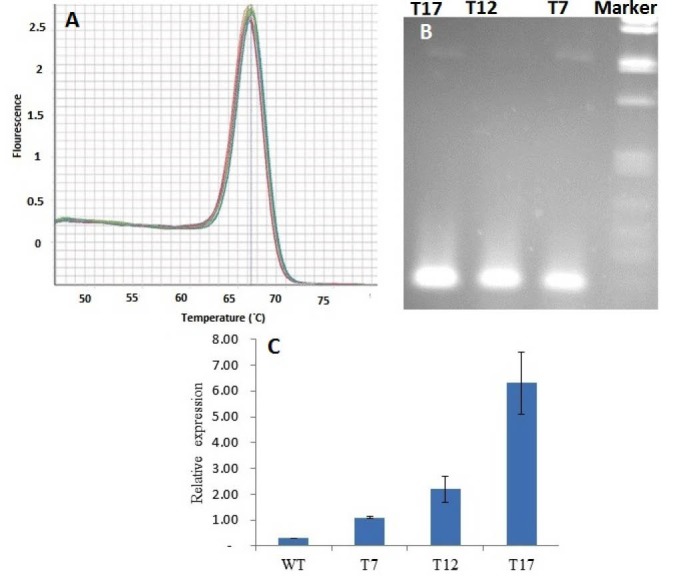
(A) Q-PCR Melt Curve analysis of the *TRR14* gene, (B) Agarose gel electrophoresis of the PCR reaction products obtained from *TRR14*-overexpressed plants and (C) Q-PCR analysis of TRR14 gene expression of the WT and TRR14-overexpressed plants (T7, T12, and T 17). TRR14 expression in over expressed lines are plotted as a ratio to the expression in the WT. Standard deviation is calculated (n=3).

To investigate the physiologic role of *TRR14*, the corresponding cDNA was integrated into a binary vector pBin19 under the control of CaMV 35S promoter and then transformed into the WT (Colombia-0 ecotype) seedlings. The full-length cDNA transformation into Arabidopsis Col-0 plants provided 20 independent lines which showed resistance to the selectable marker. Three independent transgenic lines were selected for *TRR14* gene expression analysis (T7, T12, and T17). There were not non-specific amplification and the signs of primer dimers in the PCR reactions (Figs. 1-A, B), so, gene expression analysis was valid. The transcript content of *TRR14* was more than 2 times higher in the transformed lines compared to the WT (Fig. 1-C). We used T17 transgenic line for further analysis.

### 4.2. Characterization of TRR14 Overexpressed Versus WT Plants.

**Figure 2:**
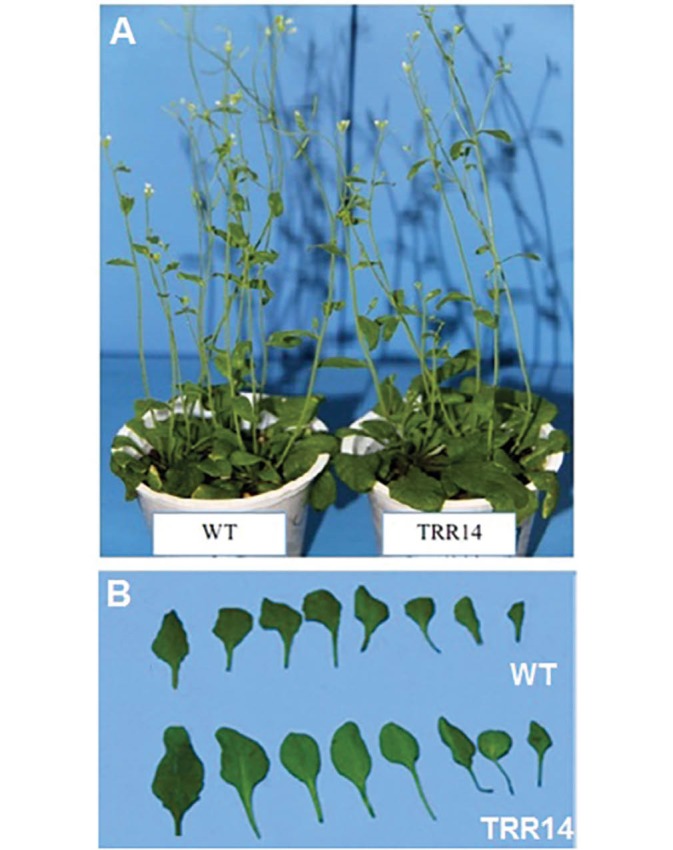
The phenotypes of the *TRR14*-overexpressed (TRR14) and the Wild-Type (WT) plants. (A) The fl ower producing *TRR14*-overexpressed plants and the control WT plants, and (B) the leaf morphology of the TRR14 and WT plants, respectively. Plants were grown in a controlled growth chamber under 16-hours-light/8-hours- dark for 5 weeks.

The *TRR14* overexpressed and WT plants showed differences in the morphology, leaf area, fresh, and dry weight when grown in soil. While there was no significant difference in the height between *TRR14* overexpressed and the WT plants (Fig. 2A), the former showed a higher leaf area compared to the WT (Fig. 2B). Furthermore, the *TRR14* overexpressed plants were not different with respect to the flowering time when compared with the WT ones. The *TRR14* overexpressed plants had significantly greater fresh and dry weights than that of the WT plants. The fresh and dry weights were 0.19 ± 0. 1 and 1.4 ± 0.01 g respectively in the *TRR1*overexpressed plants, while they were 0.8 ± 0.005 and 0.8 ± 0.09 g in the WT plants (Table 1).

**Table 1:** The effect of *TRR14*-overexpression on several growth related features and photosynthetic pigments content. Fresh weight (FW), dry weight (DW), Chlorophyll content (Chl), Chlorophyll ratio (Chl *a/b*), and carotenoid content of the Arabidopsis wild-type (WT) and *TRR14*-overexpressed (TRR14) plants that were grown under a normal condition.

	** FW (g)**	** DW (g)**	** Chl (mg.g** ^-1^ ** FW)**				**Carotenoid** **(mg.g** ^-1^ ** FW)**
			**a**	**b**	**a/b**	**Total**	
**WT**	0.8±0.09^b^	0.08±0.005^b^	4.41±1.09^a^	3.45±0.8^a^	3.29	3.29±0.77^a^	398.48±90.6^a^
**TRR14**	1.4±0.1^a^	0.19±0.01^a^	4.96±1.4^a^	3.81±0.97^a^	4.62	3.71±0.93^a^	462.95±68^a^

**Figure 3:**
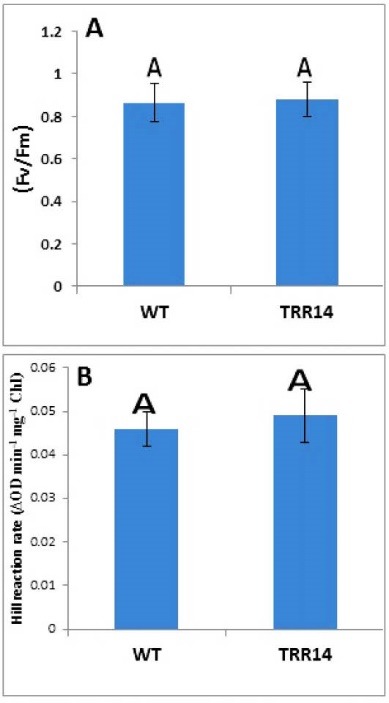
The photosynthetic parameters of the *TRR14*-overexpressed (TRR14) and the Wild-Type (WT) plants. A) Photosynthetic parameter, and B) the water oxidation capacity as measured by the rate of Hill reaction. Plants were grown in a controlled growth chamber under 16-hours-light/8-hours- dark condition (n=3).

**Figure 4:**
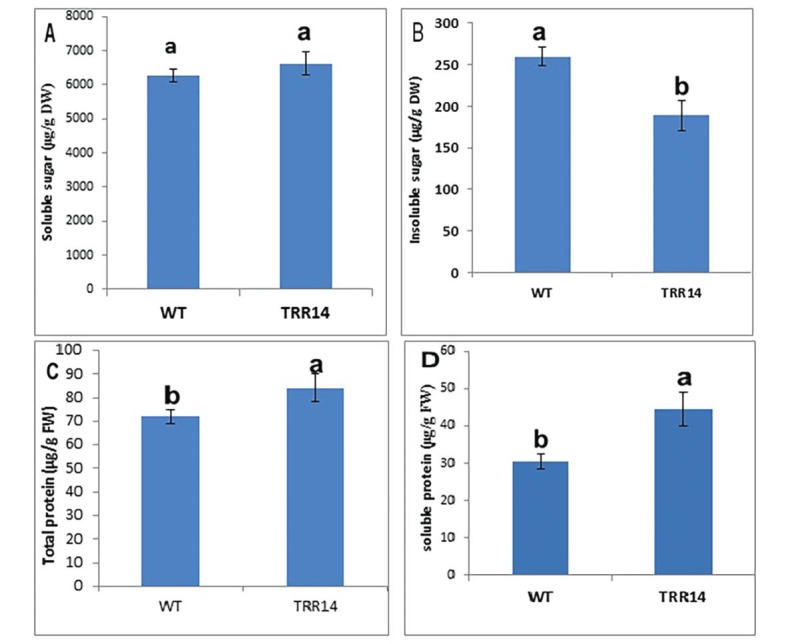
Assessment of the several biochemical factors in *TRR14*- overexpressed (TRR14) and the Wild- Type (WT) plants. A) Soluble sugar, B) Insoluble sugar, C) Total protein, and D) the Soluble proteins (n=3).

The Chl a, Chl b, total chlorophyll, carotenoid content, and Chl*a/b* ratio were similar in both *TRR14-*overexpressed and WT plants, as well (Table 1). The efficiency of the PSII (i.e. *Fv/Fm* value and the Hill reaction rate which represents the plant water oxidation capacity) was almost indifferent between *TRR14* overexpressed and the WT plants (Fig. 3B). Analysis of the carbohydrate showed that soluble sugar content was similar in both *TRR14* and WT plants (Fig. 4A). But, insoluble sugar content was significantly greater in the WT than that of the *TRR14-*overexpressed plants (Fig. 4B).

### 4.3. The Chloroplast Protein Composition of the TRR14-overexpressed and the WT Plants

The leaf materials from the *TRR14*-overexpressed and WT plants were analyzed for the soluble and total protein content in addition to chloroplast protein composition. There were significant differences in the soluble and total protein content between *TRR14-*overexpressed and WT plants. The soluble and total protein contents were lower in the WT compared to the*TRR14*-overexpressed plants (Figs. 4C, 4D).

**Figure 5:**
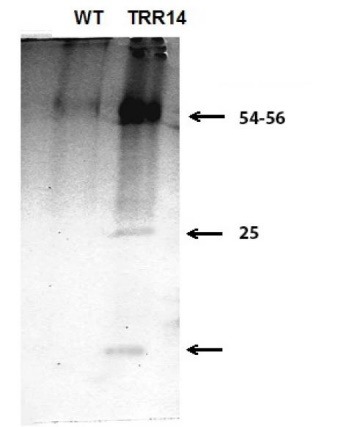
SDS-PAGE profi le of the *TRR14*- overexpressed plant (TRR14) and the WT plants. TRR14 and WT represent *TRR14*- overexpressed and the wild-type plants, respectively.

Then, isolated proteins from chloroplasts of *TRR14* overexpressed and WT plants were analyzed through SDS-PAGE. The protein band patterns of the both *TRR14* overexpressed and WT chloroplasts were similar. However, SDS-PAGE fractions detected one extra protein band corresponding to the 25 kDa in the transgenic line (Fig. 5).

### 4.4. Photorespiration is Altered by Overexpression of TRR14

**Figure 6:**
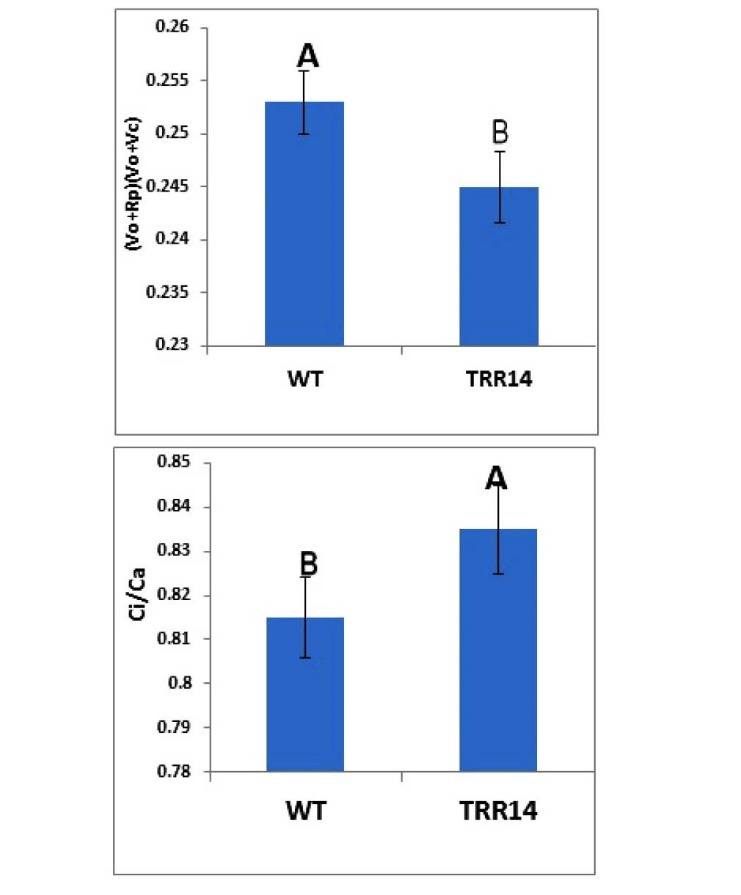
Photorespiration parameters of the *TRR14*-overexpressed (TRR14) and the Wild-Type (WT) plants. A) Ratios of the leaf internal versus the air CO_2_ concentrations, B) The measured photorespiration using the formula: (V_0_+Rp)/(V_0_+Vc). For each experiment two plants were analyzed and measurements were carried out in triplicate.

To investigate whether the *TRR14* overexpression has any effect on the plant photorespiration, we determined the CO_2_ concentrations ratios of the leaf internal spaces versus air in both WT and in the overexpressed *TRR14* plants. Measurements of the ratio of the leaf internal CO_2_(Ci) and ambient CO_2_(Ca) concentration (Ci/Ca) revealed that the gas exchange process of *TRR14* overexpressed plants is different from that of WT ones (Fig. 6A). The results further revealed that photorespiration is significantly reduced in the *TRR14-*overexpressed plants (Fig. 6B).

## 5. Discussion

The metabolism of trehalose is indispensable in the plants (30). Trehalose related metabolites are present in only very low concentrations and their role in plants is not fully understood so far (5). A minor alteration in the steady state content of T6P; the precursor of trehalose in the plants was shown to result in dramatic and pleiotropic phenotypic changes of the plants (12,31). Trehalose supplied to the growth medium of the seedlings inhibits their growth and alteration in the carbon allocation in the seedlings so that starch is preferentially accumulated in the shoots rather than the roots (32,1). However, *TRR14* overexpression provides resistance to the both growth inhibition and starch accumulation effects of T6P (1). The results obtained here have revealed that *TRR14-*overexpressed plants display a higher leaf area, fresh, and dry weight as compared to the WT plants. These results suggest that TRR14 may enhance the growth of Arabidopsis. Although the starch content of *TRR14-*overexpressed plants was lower than WT, they had greater amounts of both soluble and total proteins which may indicate an alteration in C/N metabolism of the overexpressed plants in favor of organic nitrogen production.

T6P is known to affect photosynthetic capacity per unit leaf area (12). Accordingly, photosynthesis might be a target of T6P functioning. Neither chlorophylls and carotenoids contents nor the rate of Hill reaction and photosynthetic efficiency (*Fv*/*Fm*) was appeared to be different between *TRR14-*overexpressed and WT plants (Fig. 3). These suggest that the positive effects of* TRR14* overexpression on plant growth is not related to its possible effects on light energy absorption and/or transduction through chloroplast pigment complexes and electron transport chain. The current result showed that *TRR14* overexpressed plants have a higher Ci with associated lower RP. This data revealed that overexpression of *TRR14* leads to the reduced rate of photorespiration in the plants (Fig. 6).

Naturally, some plants which display C_4_, C_3_-C_4_ intermediate, and CAM metabolisms down-regulate their photorespiration through concentrating CO_2_ at the site of carboxylation where Rubisco resides (i.e. chloroplasts, and at a larger scale the leaf’s intercellular spaces) (33). Measurement of the CO_2_ concentration in the leaf internal spaces of the *TRR14* overexpressed plants has justified their reduced rate of photorespiration. The transformed plants had significantly greater contents of the internal CO_2_ concentration than the WT plants (Fig. 6), thus, TRR14 functioning in reducing photorespiration is somehow accompanied with or through an increased CO_2_ concentration in the leaf intercellular spaces. This might explain the reason for observing a greater biomass of the transformed plants. The mechanism through which TRR14 brings about an increased concentration of intercellular CO_2_ needs further investigation. This protein might affect leaf cell development and/or metabolic compartments in favor of localized CO_2_ production.

Such a condition has been reported in *Panicum milioides* and *Moricandia arvensis,* the two C_3_-C_4_ intermediate species which their proximal chlorenchymatous cells contain high activity of glycine decarboxylase (i.e., a major CO_2_ releasing enzyme of the photorespiratory pathway) while their distal cells have a high Rubisco activity which benefit from the released CO_2_ by the proximal cells (34,35). It has been suggested that photorespiration imposes yield ceilings on most of the high-bred crops planted (33,35). However, in stress conditions such as excess light, drought, or possibly other stresses that affect guard cell functions, this process has some advantages for the C_3_ plants by ensuring their survival rather than productivity (36). Despite cloning of Arabidopsis genes which encode photorespiration pathway basic enzymes, questions regarding the regulation of photorespiration are still open and demand further scrutiny (37). Attempts to reduce photorespiration have mostly failed even though it is a major goal for crop improvement. An increased understanding of the photorespiration regulation is needed in the context of the climate change as stress-induced by drought and heat gradually increase photorespiration of the existing crops and lead to yield loss (38).

So far, the physiological function of TRR14 has largely been unknown, except the finding that *TRR14* overexpression leads to the salt and drought stress resistant (4,16). Our previous data has indicated that *TRR14* overexpressed plants had unchanged seed germination, root length, and chlorophyll content under the salt and drought stresses. In addition, the activity of several enzymes related to the scavenging reactive oxygen species like peroxidase and catalase were significantly induced in *TRR14* overexpressed under salt and drought treatment (4). The reduced photorespiration, as observed here in the *TRR14* transformed plants, implies also the reduced H_2_O_2_ production during photosynthesis which is in favor of a greater biomass production (1,4). This feature reduces plant’s energy consumption for removal of various reactive oxygen species. Further studies are needed to reveal the roles played by TRR14 in reducing plant photorespiration and tolerance to the environmental stresses. The obtained data could un-doubtfully be useful in the near future for the production of crops with greater yield performance.

It has been shown that photorespiration has an important role in the protection of photosynthetic apparatus against stress (39,40). This possibility needs further exploration since T6P and trehalose have been shown to convey stress-resistance responses that could be mediated by specific reactive oxygen signaling (41-44).

## 6. Conclusions

In conclusion, TRR14 is a novel protein important for the regulation of photorespiration, an area of research of great economic and environmental interest. With global climate change and an increasing agricultural production on marginal lands, the link between TRR14, trehalose metabolism, and photorespiration regulation is of interest for future studies.

## Funding/Support

This research has been supported by the Golestan University, Deputy of Research, and Office of Higher Education (91/71/10671).
